# Model-Free Adaptive Positioning Control of the Bidirectional Stick–Slip Piezoelectric Actuator with Coupled Asymmetric Flexure-Hinge Mechanisms

**DOI:** 10.3390/s23187795

**Published:** 2023-09-11

**Authors:** Zhenguo Zhang, Yikun Dong, Shuai Yu, Xiaohui Lu, Keping Liu

**Affiliations:** 1 School of Mechatronic Engineering, Changchun University of Technology, Changchun 130012, China; zhangzhenguo@ccut.edu.cn (Z.Z.); luxh13@ccut.edu.cn (X.L.); 2 School of Electrical and Electronic Engineering, Changchun University of Technology, Changchun 130012, China; 15568813658@163.com (Y.D.); 2202104073@stu.ccut.edu.cn (S.Y.); 3 School of Electrical and Information Engineering, Jilin Engineering Normal University, Changchun 130012, China

**Keywords:** piezoelectric stick–slip actuators (PSSAs), positioning control, model-free adaptive control (MFAC), radial basis function neural network (RBFNN)-based disturbance estimator, uncertain disturbance

## Abstract

A model-free adaptive positioning control strategy for piezoelectric stick–slip actuators (PSSAs) with uncertain disturbance is proposed. The designed controller consists of a data-driven self-learning feedforward controller and a model-free adaptive feedback controller with a radial basis function neural network (RBFNN)-based observer. Unlike the traditional model-based control methods, the model-free adaptive control (MFAC) strategy avoids the complicated modeling process. First, the nonlinear system of the PSSA is dynamically linearized into a data model. Then, the model-free adaptive feedback controller based on a data model is designed to avoid the complicated modeling process and enhance the robustness of the control system. Simultaneously, the data-driven self-learning feedforward controller is improved to realize the high-precision control performance. Additionally, the convergence of the tracking error and the boundedness of the control output signal are proved. Finally, the experimentally obtained results illustrate the advantages and effectiveness of the developed control methodology on the bidirectional stick–slip piezoelectric actuator with coupled asymmetric flexure-hinge mechanisms. The positioning error through the proposed controller reaches 30 nm under the low-frequency condition and 200 nm under the high-frequency condition when the target position is set to 100 μm. In addition, the target position can be accurately tracked in less than 0.5 s in the presence of a 100 Hz frequency.

## 1. Introduction

Piezoelectric stick–slip actuators (PSSAs) are composed of a piezoelectric stack, amplification unit, and motion unit. Since they can realize large strokes and high-precision nanometer movements [1,2], they have become the heart of high-precision driving equipment such as scanning electron microscopes [3] and other devices used in biomedicine [4]. With the development of the semiconductor industry, more and more optoelectronic devices need to be able to achieve large strokes and high-precision motion strokes, but they also need to be able to function in optical equipment; piezoelectric micro-tables have these advantages and have a large output force, allowing them to meet the corresponding requirements. However, there are some challenges related to positioning accuracy. At present, the positioning control accuracy of the PSSAs is a challenging problem since it can be affected by its inherent hysteresis nonlinearity, the complex friction–motion relationship between structures, and uncertain disturbance.

Researchers have conducted in-depth research in the field of nanomaterials [5,6], and piezoelectric actuators based on piezoelectric materials have been gradually developed. These researchers are working on new high-performance piezoelectric actuators for different uses, enabling them to meet the requirements of high-precision positioning in the integration process. The micro-positioning platform based on a stick–slip inertial piezoelectric actuator is designed in various styles according to the application requirements, where the common ones are the rotary stick–slip inertial piezoelectric actuator and the linear stick–slip inertial piezoelectric actuator. Its structural design has been maturely developed [7], and there are many new, more stable structures [8,9]. In order to apply these many new piezoelectric actuator structures, the development of intelligent control technology is becoming more and more important.

To enhance positioning accuracy and stability, the researchers have proposed a variety of control strategies. The existing control strategies of PSSAs can be divided into amplitude closed-loop control and amplitude–frequency double closed-loop control. In Ref. [10], the author designed a double closed-loop control strategy for speed and position. The first part implements the position closed-loop control through the grating displacement sensor. The second part is the speed closed-loop control that occurs through proportional–integral–derivative (PID) control. The double closed-loop control method ensures the output speed and positioning accuracy of the PSSAs. Rong W et al. [11] took the strain gauge as the displacement sensor of the PSSAs, developed a displacement prediction approach, and adopted the feedforward PID control method to improve the dynamic characteristics of the system. Theik C et al. [12] applied the PSSAs to suppress vibration and developed three distinct controllers—the PID manual adjustment, PID automatic adjustment, and PID active force controller (AFC)—to achieve an appropriate damping effect. Sliding-mode control (SMC) does not depend on an accurate mathematical model. It is a good control strategy for the complex PSSA system. Cao Y et al. [13] established a model based on autoregressive exogenous (ARX) and proposed a PID-based SMC to realize the speed and positioning control of PSSAs, to improve the adaptive ability of the controller, and to compensate for the influence of the hysteresis characteristics and internal complex friction relationship on the control accuracy. Cheng L et al. [14] proposed a model predictive controller based on the neural network approach. To this end, the macro–micro control scheme was adopted. In the micro control stage, the nonlinear approximation ability of the neural network was utilized for predictive control. This method avoids the control based on a complex fixed model and is a successful application of the intelligent control method in PSSAs. In addition to predictive control based on the neural network model, Cheng L et al. [15] proposed a predictive control methodology based on a fuzzy model. The controller aimed to establish the Takagi–Sugeno fuzzy model for the whole PSSA and design a predictive controller based on the model. Additionally, Oubellil R et al. [16] proposed a switching control strategy which adopts macro–micro control, PID control in the macro control stage, and H-∞ control in the micro control stage. The macro–micro control was capable of successful conversion via switching control. In the positioning control of PSSAs, where the control algorithm is the exception, other investigators realized the motion control of piezoelectric stick–slip actuators by controlling the charge. Martin Š et al. [17] designed an analog electronic circuit and introduced switch control technology to avoid the impact of the hardware circuit operation on the positioning accuracy. This control approach tends to be a practical application, but this is not stable enough and the corresponding cost is high. In addition to intelligent control algorithms and charge control, there are also cases in which image processing technology is applied to the motion control of PSSAs. For instance, Chen C et al. [18] designed a visual servo system to determine the end position of PSSAs in real time through specific microscopic instruments and image recognition software, so as to achieve the purpose of positioning control.

Most PSSAs controllers have been developed based on a model, and the inaccurate modeling process influences the accuracy of the positioning control. For the positioning control of PSSAs, a MFAC method based on the RBFNN disturbance estimator is proposed in this paper. The controller consists of two major parts: the data-driven self-learning feedforward controller and the model-free adaptive feedback controller. The hysteresis characteristic of PSSAs is then compensated for by a data-driven self-learning feedforward controller. Simultaneously, the model-free adaptive feedback controller is introduced to enhance the robustness of the controller. For the problem of the uncertain disturbance affecting the positioning accuracy, an RBFNN disturbance estimator is also introduced into the model-free adaptive controller, and the corresponding stability analysis is also provided and explained. Finally, the experimental verification is carried out on a bidirectional stick–slip piezoelectric actuator with coupled asymmetric flexure-hinge mechanisms. The experimentally observed results verify the effectiveness of the proposed control strategy.

## 2. The Working Principle and Controlled Requirements of the Platform

### 2.1. Working Principle

**Figure 1:**
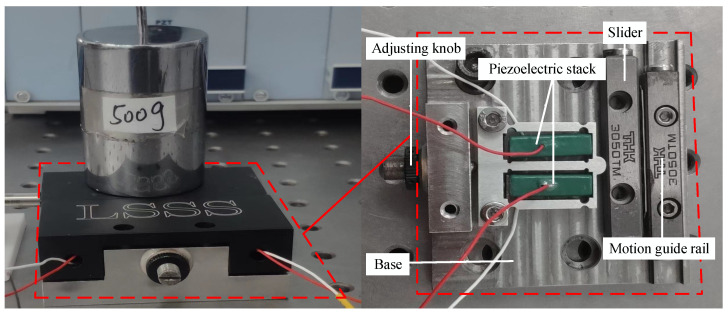
Pictures of the controlled physical platform.

As shown in Figure 1, a bidirectional PSSA with coupled asymmetric flexure-hinge mechanisms [19] was employed, and the part marked in the red box represents its structural appearance and a schematic representation of its internal structure. The PSSA consists of an asymmetric flexure hinge, two piezoelectric stacks, a moving guide rail, and a base. The actuator exploits the coupled asymmetric flexure-hinge mechanism and symmetrical indenter to produce controllable tangential displacement. The adjusting knob on the base is used to adjust the preload between the asymmetric flexure-hinge press head and the slide block.The type of piezoelectric stack used in this paper is AE0505D16, the safe driving voltage range is 0–150 V, the size is 5 mm × 5 mm × 20 mm, and it is mechanically composed of multiple layers of piezoelectric ceramic sheets in a series. The material of the asymmetric flexure-hinge mechanism is 7075 aviation aluminum alloy, the density is 2810 kg/m^3^, the Young’s modulus is 7.17 × 10${}^{10}$ N/m^2^, and the Poisson’s ratio is 0.33.

**Figure 2:**
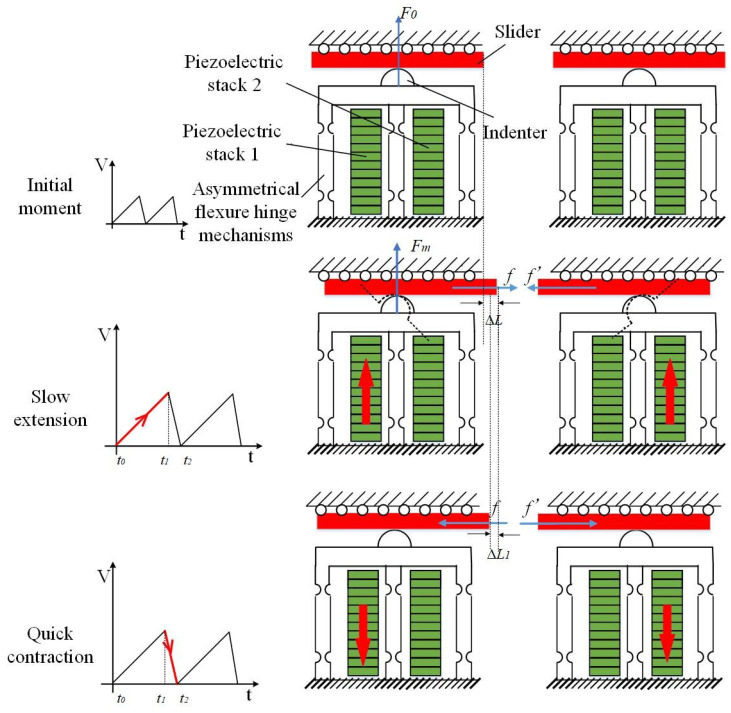
Motion principle of the bidirectional PSSA.

Figure 2 demonstrates the motion principle of the bidirectional PSSA with coupled asymmetric flexure-hinge mechanisms. Due to the asymmetric stiffness of the flexure hinge, both longitudinal and tangential displacements are produced by the indenter when the left or right piezoelectric stacks are distinctly stretched. At the initial moment, the exerted force on the slider due to the indenter is denoted by $F_{0}$, which is the given preload (i.e., initially applied force). When the control signal acts on the left stacks, the slider moves to the right. On the contrary, when it acts on the right stacks, the slider moves to the left. The slider realizes continuous stick–slip movement under the action of a sawtooth wave.

Sticking stage: From time $t_{0}$ to time $t_{1}$, the applied voltage rises gradually. When the voltage acts on the left piezoelectric stacks, the slider and the indenter remain relatively stationary during the movement in the tangential direction. The force generated in the longitudinal direction grows from $F_{0}$ to its maximum value (i.e., $F_{m}$) at time $t_{1}$. The slider is subjected to a frictional force from the left to the right. By applying the driving voltage to the right piezoelectric stacks, the slider will be subjected to a frictional force ($f^{\prime }$) to the left. The slider moves forward by a distance of ΔL at time $t_{1}$ relative to time $t_{0}$.

Sliding stage: From $t_{1}$ to $t_{2}$, the voltage level drops abruptly, the indenter rapidly shrinks to the position at moment $t_{0}$ in the tangential direction, and a relative movement between the indenter and the slider occurs. Due to the existence of inertia, the slider produces a backsliding displacement $\Delta L_{1}$.

### 2.2. Control Problem Statement

For the bidirectional PSSA with coupled asymmetrical flexure-hinge mechanisms, the single-step displacement is $\Delta y\left(t\right)=\Delta L-\Delta L_{1}$, where Δy(t) denotes the single-step displacement. The PSSA system can be equivalent to the nonlinear input–output mapping displayed by the following relation:

(1) ∑:y(t)=H(u(t))

(2) ∑:y(k)=H(u(k))

where u(t) and y(t), in order, are the input and output of the nonlinear system, and *H* represents the nonlinear operator. Equation (2) represents its discrete form. We hope to be able to control the input voltage, u(t), via an appropriate closed-loop control, so that the output, y(t), reaches the desired position.

The bidirectional stick–slip piezoelectric actuator with an asymmetric flexure hinge can achieve bidirectional motion by controlling the driving voltage of two piezoelectric stacks through an open-loop scheme. To realize the adaptive control of the platform, sensors and the MFAC algorithm are introduced to realize the precise positioning control of the PSSA. In addition, because of the complex uncertainties of the system model and the influence of external and internal disturbances on the positioning accuracy, a model-free adaptive data-driven controller based on RBFNN estimation is designed, which enables the platform to achieve accurate positioning.

## 3. Design and Stability Analysis of the Model-Free Adaptive Positioning Controller

### 3.1. Design of the Model-Free Adaptive Positioning Controller

The proposed model-free adaptive controller consists of two basic parts: a feedforward controller and a feedback controller. First, the model-free adaptive feedback controller is designed. The analysis and design are carried out in a discrete case [20].

(3) $$y\left(k+1\right)=f(y\left(k\right),\ldots ,y\left(k-n_{y}\right),u\left(k\right),\ldots u\left(k-n_{u}\right))$$

(4) $$\left|y\left(k_{1}+1\right)-y\left(k_{2}+1\right)\right|\leq b\left|u\left(k_{1}\right)-u\left(k_{2}\right)\right|$$

The discrete form of the single-input single-output nonlinear system is expressed by Equation (3), where y(k)∈R, u(k)∈R, and *n* is a positive integer. Further, y(k) and u(k) represent the output displacement and the input voltage of the PSSA at the current time.

The application of a model-free adaptive controller should meet two prerequisites. As shown in Equation (3), under all *k* values (except individual *k* values), the partial derivative of the *f* function with respect to the $\left(n_{y}+2\right)$th variable is continuous. Then, the system satisfies the generalized Lipschitz condition, as displayed by Equation (4). It is clear that the PSSA system is satisfied. After meeting the above two conditions, the dynamically linearized mathematical model of the PSSA can be derived. The displacement increment is given by Equation (5), and the system input–output model is presented by Equation (6):

(5) $$\Delta y\left(k+1\right)=f_{c}\left(k\right)\Delta u\left(k\right)$$

(6) $$y\left(k+1\right)=y\left(k\right)+f_{c}\left(k\right)\Delta u\left(k\right)$$

where Δy(k+1) denotes the displacement increment of the mathematical model at the next time, Δu(k) represents the input voltage difference between the current time and the previous time and $f_{c}\left(k\right)$ stands for the pseudo partial derivative of the current moment (k), which is indicated by the pseudo partial derivative estimation method. Before designing the pseudo partial derivative, an error cost function for evaluating the motion quality of the PSSA is proposed. The error cost function can be expressed by:

(7) $$J\left(u\left(k\right)\right)={\left|y(k+1)-y(k+1)\right|}^{2}+\lambda {\left|u(k)-u(k-1)\right|}^{2}$$

where y(k+1) represents the expected output displacement of the PSSA and λ denotes the weight factor used to adjust the change rate of the input voltage. According to Equations (6) and (7), the expression of $u_{1}\left(k\right)$ can be obtained as follows:

(8) $$u_{1}\left(k\right)=u\left(k-1\right)+\frac{f_{c}\left(k\right)}{\lambda +{\left|f_{c}\left(k\right)\right|}^{2}}\left(y(k+1)-y(k)\right)$$

In Equation (8), a step factor (γ) can be introduced to adjust the voltage increment; as a result, the control strategy of the PSSA can be obtained in the following form:

(9) $$u_{1}\left(k\right)=u\left(k-1\right)+\frac{\gamma f_{c}\left(k\right)}{\lambda +{\left|f_{c}\left(k\right)\right|}^{2}}\left(y(k+1)-y(k)\right)$$

Of these, the function $f_{c}\left(k\right)$ is designed by the pseudo partial derivative estimation method. Before proceeding with this, another error cost function, as given by Equation (10), is proposed:

(10) $$J\left(f_{c}\left(k\right)\right)={\left|y\left(k\right)-y\left(k-1\right)-f_{c}\left(k\right)\Delta u\left(k-1\right)\right|}^{2}+m{\left|f_{c}\left(k\right)-\hat{f_{c}}\left(k-1\right)\right|}^{2}$$

where *m* represents the weight factor and $\hat{f_{c}}\left(k-1\right)$ denotes the estimated value of the pseudo partial derivative. Using Equations (6) and (10), the expression of $\hat{f_{c}}\left(k\right)$ can be obtained, and the second step factor (*h*) is introduced to arrive at the new estimation formula of the pseudo partial derivative $\hat{f_{c}}\left(k\right)$:

(11) $$\hat{f_{c}}\left(k\right)=\hat{f_{c}}\left(k-1\right)+\frac{h\Delta u(k-1)}{m+{\left|\Delta u(k-1)\right|}^{2}}\left(\Delta y\left(k\right)-\hat{f_{c}}\left(k-1\right)\Delta u\left(k-1\right)\right)$$

Next, a reset algorithm should be introduced if $\hat{f_{c}}\left(k\right)<c$ or $\left|\Delta u(k-1)\right|\leq c$, where *c* denotes a given constant value, or if $sign\left(\hat{f_{c}}\left(k\right)\right)\neq sign\left(\hat{f_{c}}\left(1\right)\right)$ or $\hat{f_{c}}\left(k\right)=\hat{f_{c}}\left(1\right)$ is then established, where 0<h≤2 and m>0.

By introducing Equation (11) to Equation (9), the feedback control strategy of the PSSA can be derived as:

(12) $$\begin{array}{c} u_{1}\left(k\right)=u_{1}\left(k-1\right)+\frac{\gamma \left[\hat{f_{c}}\left(k-1\right)+\frac{h\Delta u(k-1)}{m+{\left|\Delta u(k-1)\right|}^{2}}\left(\Delta y\left(k\right)-\hat{f_{c}}\left(k-1\right)\Delta u\left(k-1\right)\right)\right]}{\lambda +{\left|f_{c}\left(k\right)\right|}^{2}}\\ \left(y(k+1)-y(k)\right) \end{array}\;$$

(13) $$u_{2}\left(k\right)=W\left(k\right)R\left(k\right)$$

(14) $$W\left(k\right)=[w_{0}\left(k\right),w_{1}\left(k\right),\cdots ,w_{N}\left(k\right)]$$

(15) $$R\left(k\right)={\left[r(k),r(k-1),\cdots ,r(k-N)\right]}^{T}$$

The feedforward control strategy is mathematically represented by Equation (13) [21], where $u_{2}$ indicates the feedforward controller output and $u_{1}$ indicates the feedback controller output. W(k) denotes the learning parameter vector of the feedforward controller, and $R\left(k\right)$ is the parameter vector of the desired position, $R\left(k\right)=y*\left(k\right)$. W(k) is updated by the steepest descent algorithm in the following form:

(16) $$W\left(k\right)=W\left(k-1\right)+\mu \left(k-1\right)R^{T}\left(k-1\right)e\left(k-1\right)$$

For the PSSA, the model-free adaptive positioning control strategy based on the data drive can be stated using:

(17) $$u\left(k\right)=u_{1}\left(k\right)+u{}_{2}\left(k\right)$$

(18) $$\begin{array}{c} u\left(k\right)=u\left(k-1\right)+\frac{\gamma \left[\hat{f_{c}}\left(k-1\right)+\frac{h\Delta u(k-1)}{m+{\left|\Delta u(k-1)\right|}^{2}}\left(\Delta y\left(k\right)-\hat{f_{c}}\left(k-1\right)\Delta u\left(k-1\right)\right)\right]}{\lambda +{\left|f_{c}\left(k\right)\right|}^{2}}\\ \left(y(k+1)-y(k)\right)+W\left(k\right)R\left(k\right) \end{array}$$

**Figure 3:**
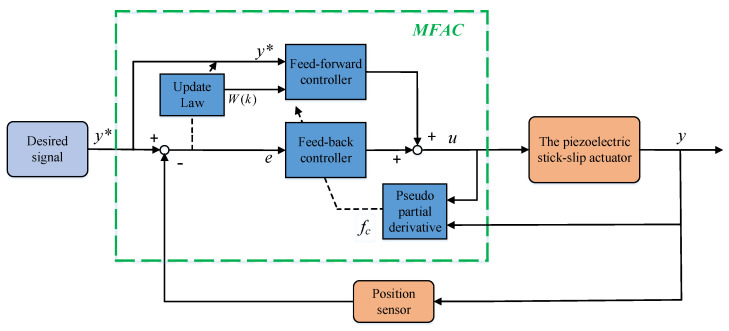
Schematic block diagram of the MFAC control system.

The control system block diagram based on the MFAC algorithm is illustrated in Figure 3.

In the movement process of the PSSA, the output value of the controller would be unstable due to the uncertain disturbance; hence, the positioning of the PSSA would not be accurate enough. Therefore, in the dynamically linearized data model, we define the generalized perturbation of the system as $y_{e}\left(k\right)$, such that $\left|y_{e}\left(k\right)\right|\leq M$, in which *M* denotes a normal number. As shown in Equation (19), a 1-3-1 layer RBFNN could be utilized to identify it online. The principle is illustrated in Equation (20):

(19) $$y\left(k+1\right)=y\left(k\right)+f_{c}\left(k\right)\Delta u\left(k\right)+y_{e}\left(k\right)$$

(20) $$y_{e}\left(k\right)=w{\left(k\right)}^{T}h\left(k\right)+\varepsilon =\hat{w}{\left(k\right)}^{T}h\left(k\right)+\varepsilon$$

where w(k) denotes the weight of the RBFNN and h(k) represents the activation function of the RBFNN. The estimation error of the disturbance is indicated as the input of the neural network estimator, ε denotes the estimated error between the estimated disturbance under the expected condition and the actual disturbance and $\hat{w}\left(k\right)$ represents the actual estimated weight. To determine $\hat{w}\left(k\right)$, the error index of the RBFNN approximation is defined by:

(21) $$E\left(k\right)=1/2\varepsilon^{2}$$

where ε can be evaluated using Equations (19) and (20). According to the gradient descent methodology, the update to $\hat{w}\left(k\right)$ can be adjusted as follows:

(22) $$\hat{w}\left(k\right)=\hat{w}\left(k-1\right)+\eta \frac{\partial E}{\partial \hat{w}}$$

Similarly, according to the design methods of Equations (9) and (11), the control law with the disturbance estimation and the pseudo partial derivative estimation can be obtained in the same way. The control strategy of the PSSA can be obtained, as displayed in Equation (23), and the pseudo partial derivative can be estimated, as given by Equation (24):

(23) $$u\left(k\right)=u\left(k-1\right)+\frac{\gamma f_{c}\left(k\right)}{\lambda +{\left|f_{c}\left(k\right)\right|}^{2}}\left(y*\left(k+1\right)-y\left(k\right)-\hat{w}{\left(k\right)}^{T}h\left(k\right)\right)$$

(24) $$\hat{f_{c}}\left(k\right)=\hat{f_{c}}\left(k-1\right)+\frac{h\Delta u(k-1)}{m+{\left|\Delta u(k-1)\right|}^{2}}\left(\Delta y\left(k\right)-\hat{w}{\left(k\right)}^{T}h\left(k\right)-\hat{f_{c}}\left(k-1\right)\Delta u\left(k-1\right)\right)$$

**Figure 4:**
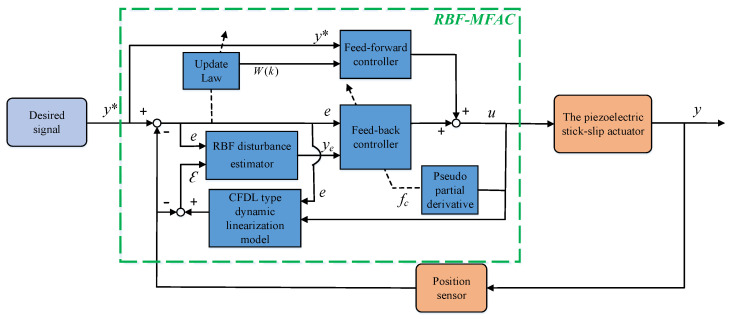
The block diagram of the MFAC system based on the RBFNN estimation.

By introducing Equation (24) to Equation (23), a new control strategy of the PSSA can be obtained. The control system block diagram of the MFAC algorithm based on the RBFNN estimation is illustrated in Figure 4.

### 3.2. Stability Analysis

This section aims to discuss the stability of the designed controller. The asymptotic stability of the controller is proved by showing that the error e(k) demonstrates convergence and the output u(k) demonstrates boundedness. This is achieved by showing the following:

(a) e(k) and $\Delta W\left(k\right)$ are convergent;
(b) ${\hat{f}}_{c}\left(k\right)$ and $u\left(k\right)$ are bounded.

When the bidirectional PSSA with coupled asymmetric flexure-hinge mechanisms experiences stick–slip motion, the backward motion occurs in an instant. According to Ref. [22], the nonlinear system of PSSAs is a dissipative system with finite gain. It is sufficient to use the relation $y\left(k\right)=\beta u\left(k\right)$ instead of $y\left(k\right)=H\left(k\right)$. First, prove $e\left(k\right)$ demonstrates convergence. We define a positive function:

(25) $$V\left(k\right)=e^{2}\left(k-1\right)$$

(26) $$\Delta V\left(k\right)=e^{2}\left(k-1\right)-e^{2}\left(k-2\right)=\Delta e\left(k-1\right)\left[2e(k-1)-\Delta e(k-1)\right]$$

According to Ref. [23], assume:

(27) $$\frac{\Delta e(k-1)}{\Delta W(k-1)}\approx \frac{\partial e(k-1)}{\partial W(k-1)}=\frac{\partial (r_{1}\left(k-1\right)-\beta W\left(k-1\right)\varphi \left(k-1\right))}{\partial W(k-1)}=-\beta \varphi \left(k-1\right)$$

It can be obtained from the weight update rate shown in Equation (16):

(28) $$\Delta W\left(k\right)=\mu_{1}\left(k-1\right)\varphi^{T}\left(k-1\right)e\left(k-1\right)$$

By introducing Equation (28) into Equation (27), we obtain:

(29) $$\Delta e\left(k-1\right)=-\gamma \Delta W\left(k-1\right)\varphi \left(k-1\right)=-\mu_{1}\left(k-1\right)\gamma \varphi^{T}\left(k-1\right)\varphi \left(k-1\right)e\left(k-1\right)$$

By introducing Equation (29) into Equation (26), we obtain:

(30) $$\Delta V\left(k\right)=-\mu_{1}\left(k-1\right)\phi \left(k-1\right)e^{2}\left(k-1\right)\left(2-\mu_{1}\left(k-1\right)\phi \left(k-1\right)\right)$$

where $\phi \left(k-1\right)=\gamma \varphi^{T}\left(k-1\right)\varphi \left(k-1\right)\geq 0$. When $2-\mu_{1}\left(k-1\right)\phi \left(k-1\right)>0$, we obtain $\mu_{1}\left(k-1\right)<2\phi^{-1}\left(k-1\right)$. In this case, ΔV(k)<0. This proves that $\lim_{k\to \infty }\;e\left(k\right)=0$.

Then, we prove that $W\left(k\right)$ demonstrates convergence.

Because the weight value a of the feedforward controller is updated via the gradient descent method, it can be obtained using:

(31) $$\Delta W\left(k\right)=W\left(k\right)-W\left(k-1\right)=\mu_{1}\left(k-1\right)\varphi^{T}\left(k-1\right)e\left(k-1\right)$$

Because e(k) is convergent, $\mu_{1}\left(k-1\right)$ is a constant parameter, and $\varphi^{T}\left(k-1\right)$ is the bounded expected trajectory. This proves that $\lim_{k\to \infty }\;\Delta W\left(k\right)=0$. Due to W(k) being convergent, φ(k−1) is the bounded expected trajectory, which proves that $u_{1}$ is also bounded.

If $\left|{\hat{f}}_{c}\left(k\right)\right|\leq e$, $\left|\Delta u\left(k-1\right)\right|\leq \tau$ or ${\hat{f}}_{c}\left(k\right)\neq sign\left({\hat{f}}_{c}\left(k\right)\right)$, *e* and τ are constants. It can be directly found that ${\hat{f}}_{c}\left(k\right)$ is bounded in the controller [24].

Because $y*\left(k\right)$ is constant, the convergence of $e\left(k\right)$ means that $y\left(k\right)$ is also bounded.

(32) $$\left|\Delta u\left(k\right)\right|=\left|\frac{\gamma {\hat{f}}_{c}\left(k\right)\left(y*\left(k\right)-y\left(k\right)\right)}{\lambda +{\left|{\hat{f}}_{c}\left(k\right)\right|}^{2}}\right|=\left|\frac{\gamma {\hat{f}}_{c}\left(k\right)}{\lambda +{\left|{\hat{f}}_{c}\left(k\right)\right|}^{2}}\right|\left|e\left(k\right)\right|$$

Because $\lambda +{\left|{\hat{f}}_{c}\left(k\right)\right|}^{2}={\left(\sqrt{\lambda }\right)}^{2}+{\left|{\hat{f}}_{c}\left(k\right)\right|}^{2}\geq 2\lambda {\hat{f}}_{c}\left(k\right)$, so

(33) $$\left|\frac{\gamma {\hat{f}}_{c}\left(k\right)}{\lambda +{\left|{\hat{f}}_{c}\left(k\right)\right|}^{2}}\right|\left|e\left(k\right)\right|\leq \left|\frac{\gamma {\hat{f}}_{c}\left(k\right)}{2\sqrt{\lambda }{\hat{f}}_{c}\left(k\right)}\right|\left|e\left(k\right)\right|\leq \left|\frac{\gamma }{2\sqrt{\lambda }}\right|\left|e\left(k\right)\right|$$

This proves that $u\left(k\right)$ is bounded.

## 4. Experimental Verification and Results

### 4.1. Experimental System

**Figure 5:**
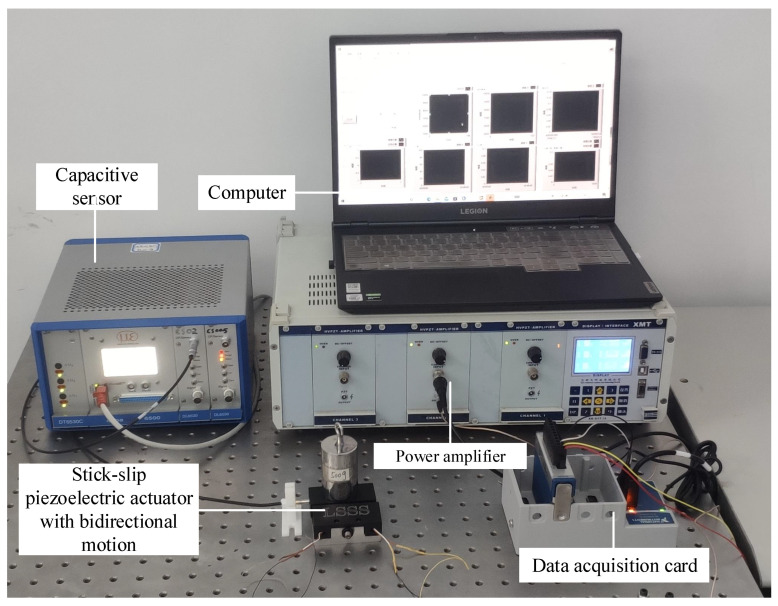
The experiment platform of the PSSA positioning control.

The experimental platform of this paper is presented in Figure 5. It includes a power amplifier (xe-500-c, Harbin Core Tomorrow Science and Technology Co., Ltd. of China, Harbin, China), a data acquisition card (NI cdaq-9174) and a 16-bit analog voltage output module (NI 9263), a capacitive non-contact displacement sensing system (DT6530C-CS005, Micro-Epsilon, where the resolution is 1 nm), a computer, an optical vibration isolation platform (63-9015 m, America), and a bidirectional stick–slip piezoelectric actuator with a coupled asymmetric flexure-hinge mechanism. The function of the data acquisition card and analog voltage output module is to output the analog sawtooth signal adjusted by the intelligent control algorithms to the power amplifier, and the amplified driving signal acts on the actuator. The capacitive sensor is responsible for the real-time detection and acquisition of the position information from the end of the slider. Through communication with the computer, the collected data can be transmitted to the computer control system.

Since the control principle of the bidirectional PSSA is the same in two directions, the experimental content and analysis of the stack actuator are conducted in the direction of unilateral motion. In this experiment, the single-step displacement of the PSSA is adjusted by the control algorithm, so that the single-step displacement gradually decreases to zero when the actuator approaches the target position, and the accuracy control of the PSSA is appropriately realized. The output range of the controller is limited to 0–6 V, and the maximum output voltage of the power amplifier amplification is set not to exceed 100 V. The drive signal frequency is set by assignment.

### 4.2. Control Experiment Verification under Different Frequencies

In order to show the effectiveness of the disturbance estimation, this paper divides the experiment into two groups for comparison. First, in the no-load condition, the control experiments of the MFAC and the MFAC based on the RBFNN disturbance estimation for determining the positioning control accuracy of the PSSA are verified. Before the formal experiment, the controller tracks the data collected under the open-loop drive to fit the model curve to determine the initial value of the self-learning parameters of the feedforward controller, where the controller parameters are h=0.22, m=9.31, γ=0.721, and λ=0.148. Let us set the sampling rate to 100 Hz for all the experiments, so that the actual displacement curve collected by the upper computer (including the closed-loop control algorithm) is the same as that of the open-loop direct drive (no control algorithms). The experimental target position is set to be equal to 100 μm, ensuring that the locking force based on the asymmetric flexure hinge is constant (i.e., the initial pressure of the indenter and the slider remains unchanged), and the value of the preload is set to 2.5 N. The comparison experiments are carried out at the sawtooth frequency levels of 1, 5, 10, 20, 40, 60, 80, and 100 Hz.

**Figure 6:**
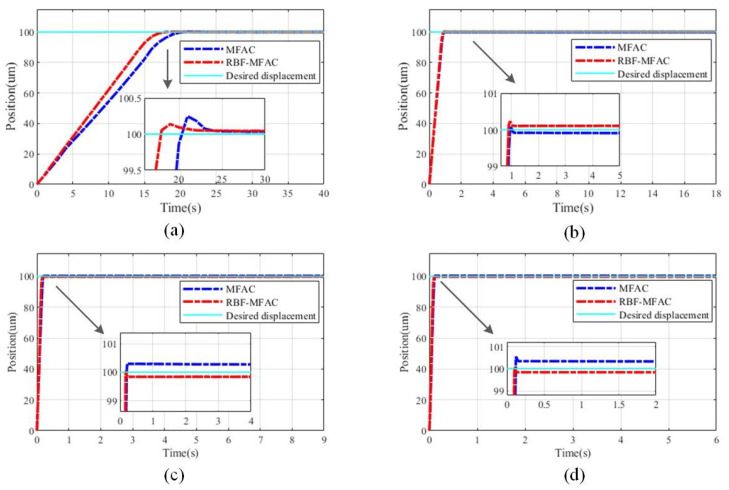
Comparison of the control effects under the action of various frequencies: (**a**) 1 Hz, (**b**) 20 Hz, (**c**) 60 Hz, (**d**) 100 Hz.

After completing the adjustment of the hardware platform, a comparison of the experimental effects is obtained by adjusting the parameters. In Figure 6, the control experiment effect comparisons of the two controllers are demonstrated at 1, 20, 60, and 100 Hz.

In the representation of the control effect, the concept of the average error is introduced, and the error of sampling points tends to be averaged to represent the control error of the controller at the control frequency. The error pairs of control effects under no-load conditions are presented in Table 1. The control error of the MFAC algorithm method based on the RBFNN disturbance estimation is lower than that of the MFAC adaptive algorithm for most considered frequencies. Under the low-frequency condition, the vibration generated by itself is almost small, which has a trivial impact on the final positioning accuracy of the PSSA. The disturbance essentially comes from the external environment. The estimation of self-vibration by the RBFNN has no noticeable impact on the controller. However, under high-frequency input, the external disturbance seriously affects the control accuracy. The introduction of the RBFNN disturbance estimator could effectively compensate for the control error.

### 4.3. Control Experiment Verification under the Action of Different Loads

In the case of no-load conditions, the two proposed control methods are appropriately verified. Since the bidirectional PSSA with a coupled asymmetric flexure hinge has a load-bearing function in practical applications, the control contrast experiment with load is set up in the experiment. The previous experiment revealed that the PSSA is stable under low-frequency control. Therefore, the control experiment analyses of various weights are carried out under the sawtooth frequency of 10 Hz. The driving force is related to voltage, frequency, locking force, and other factors. Within the maximum voltage of 100 V and a frequency of 100 Hz, the maximum load capacity under the current locking force can reach 700 g. In the present paper, the load value of the control experiment under load conditions is set as 50, 100, 200, 300, 400, 500, 600, and 700 g. In the experiment, the load function is realized by adding weights. The expected position and locking force of the mechanism are the same as those under no-load conditions.

**Figure 7:**
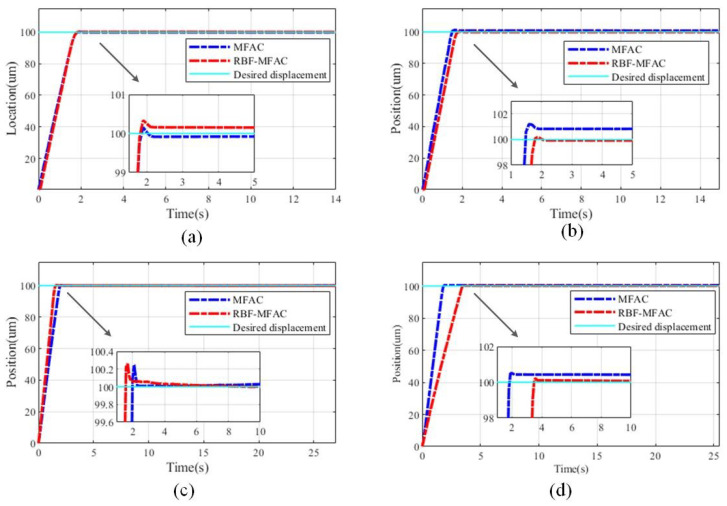
Comparison of the control effects under the action of various load levels: (**a**) 50 g, (**b**) 200 g, (**c**) 400 g, (**d**) 700 g.

Figure 7 demonstrates the two control experimental effects at the load quality levels of 50, 200, 400, and 700 g. The control error of the controller is also stated by evaluating the average error value under the control stability condition. The specific control error effect is presented in Table 2.

The obtained results in Table 2 demonstrate that when the load is exerted on the slider, the instability of the drive system magnifies, and the general control error grows. The robustness of the MFAC based on the RBFNN disturbance estimation controller is stronger than that of the MFAC composite controller. When more than two weights are added with individual mass values, the stability of the drive system could be seriously affected by the high-frequency movement. For instance, under the action of 200 g and 300 g load masses, multiple small mass weights are added. In the process of movement, the instability of both the driving platform and the load platform is enhanced. The resulting control error is massive. The MFAC based on the RBFNN disturbance estimation controller is more apparent. The RBFNN achieves good estimation of and compensation for its own vibration and lessens the system control error.

From Figure 6 and Figure 7, it can be seen that the introduction of the RBFNN disturbance estimator has a great impact on the positioning accuracy both under and without load conditions. In addition to the positioning accuracy performance, the positioning speed of the PSSA with an asymmetric flexible hinge is chiefly related to the sawtooth frequency set in the experiment. Based on Figure 6, the times taken to reach the desired positions at 1, 20, and 100 Hz are within 20, 1.5, and 0.2 s, respectively; however, the positioning accuracy will be reduced in the case of 100 Hz. Under the experimental condition with load, the driving frequency is fixed. With the increase in load, the positioning speed may also be affected; nevertheless, the driving frequency is the main factor affecting the positioning speed. During the experiment, because the output voltage regulated by the closed-loop controller has high accuracy, when the error is extremely small, the output value of the controller is also very small. Therefore, the driving force of the actuator is insufficient, resulting in a small steady-state error. In terms of the overall results, the advantages of the proposed controller are well proved.

## 5. Conclusions

In this paper, an adaptive control method of MFAC was proposed for a bidirectional PSSA with an asymmetric flexure hinge, which is characterized by imprecise modeling, poor robustness under open-loop control, external disturbance, and internal vibration affecting the positioning accuracy. In order to compensate for the influence of external disturbance and vibration on the positioning control accuracy, a compound control method of MFAC based on the RBFNN disturbance estimation was established. The stability of the controller was then proven. Finally, a comparative experiment was carried out on the experimental platform. The experiment verified the comparison of the control effect under the conditions with and without loads. The obtained results reveal that the control effect of the two controllers is better than that of the open-loop control, which avoids the process of accurate modeling and enhances the stability of the control system. Based on the RBFNN disturbance estimation, the MFAC adaptive control approach can effectively compensate for the influence of disturbance on the control accuracy by estimating disturbance and vibration, and the positioning control error is 30 nm under a large-stroke motion. Using the MFAC method based on disturbance estimation for a stick–slip inertial piezoelectric actuator with an asymmetric flexible hinge, this paper realizes a load motion with a large stroke and high precision, providing a new theoretical and experimental basis for the manufacturing and development of semiconductor equipment components. Since we used only a single experimental environment in this study and there were limitations to the experimental equipment, in future research, we will examine the adaptive algorithm with stronger robustness to determine the operating conditions of the actuator in an actual complex application environment, and we will improve the equipment, sampling frequency, and computer computing ability to further improve the control accuracy.

## Figures and Tables

**Table 1:** Error comparison diagram in the case of no-load conditions.

Frequency (Hz)	MFAC Composite Controller (Unit: μm)	MFAC Based on RBFNN Disturbance Estimation Controller (Unit: μm)
1	30.600	28.720
5	37.700	33.107
10	47.953	36.932
20	118.188	94.71
40	167.960	159.185
60	268.135	147.086
80	264.185	186.134
100	293.288	159.655

**Table 2:** Error comparison diagram for various loads.

Load Quality (g)	MFAC Composite Controller (Unit: μm)	MFAC Based on RBFNN Disturbance Estimation Controller (Unit: μm)
50	0.121	0.084
100	0.508	0.201
200	1.123	0.071
300	1.073	0.497
400	0.055	0.046
500	0.311	0.094
600	0.379	0.253
700	0.463	0.051
